# In situ simulation training for parental presence during critical situations in PICU: an observational study

**DOI:** 10.1007/s00431-022-04425-8

**Published:** 2022-03-12

**Authors:** Alice Bordessoule, Cristina Felice-Civitillo, Serge Grazioli, Francisca Barcos, Kevin Haddad, Peter C. Rimensberger, Angelo Polito

**Affiliations:** Pediatric and Neonatal Intensive Care Unit, Department of Pediatrics, Gynecology and Obstetrics, University Hospitals of Geneva, University of Geneva, Rue Willy Donze 6, 1205 Geneva, Switzerland

**Keywords:** In situ simulation, Team training, Patient- and family-centered care, Healthcare professionals stress, Family-witnessed resuscitation

## Abstract

Family presence during invasive procedures or cardiopulmonary resuscitation (CPR) is a part of the family-centered approach in pediatric intensive care units (PICUs). We established a simulation program aiming at providing communication tools to healthcare professionals. The goal of this study was to evaluate the impact of this program on the stress of PICU professionals and its acceptance. An observational study of a simulation program, with questionnaire, was used to measure pre- and post-simulation stress and the degree of satisfaction of the participants. PICU of Geneva Children’s Hospital, Switzerland. Forty simulations with four different simulation scenarios and various types of parental behavior, as imitated by professional actors, were completed during a 1-year period. Primary outcomes were the difference in perceived stress level before and after the simulation and the degree of satisfaction of healthcare professionals (nursing assistants, nurses, physicians). The impact of previous experience with family members during critical situations or CPR was evaluated by variation in perceived stress level. Overall, 201 questionnaires were analyzed. Perceived stress associated with parental presence decreased from a pre-simulation value of 6 (IQR, 4–7) to 4 (IQR, 2–5) post-simulation on a scale of 1–10. However, in 25.7% of cases, the individually perceived post-simulation stress level was higher than the pre-simulation one. Satisfaction of the participants was high with a median of 10 (IQR, 9–10) out of 10.

*Conclusions*: A simulation program helps reduce PICU team emotional stress associated with the presence of family members during critical situations or CPR, and is welcomed by PICU team members.

**Table:** 

**What is Known:** *• Family presence during cardiopulmonary resuscitation (CPR) or critical situations is a part of the family-centered approach in pediatric intensive care.* *• The benefits for the family have been already demonstrated. However, this policy is still controversy among healthcare professionals.*	
**What is New:** *• A simulation program seeking to provide skills focused on family presence management in the PICU is useful to reduce stress and was well accepted by participants.* *• It might become an indispensable training intervention for the implementation of a PICU policy to allow family presence during CPR or other critical situations.*

**Supplementary information:**

The online version contains supplementary material available at 10.1007/s00431-022-04425-8.

## Introduction

Patient- and family-centered care is an approach that underlines the essential role of family in patient healthcare and promotes a beneficial collaboration with healthcare professionals. Parents are usually willing to be present and participate in the decision-making when invasive procedures are performed on their child [1]. For this reason, family members’ presence during invasive procedures and cardiopulmonary resuscitation (CPR) has been proposed as an essential aspect of a family-centered approach to pediatric intensive care (PICU). Family presence (FP) is thought to increase in patients’ comfort during critical situations, help with the bereavement process [2, 3], help the family know that everything possible was done, and provide family members with the feeling that they had supported their child [4–7]. Family members who were present during CPR were found to have lower anxiety and depression scores, fewer disturbing memories, and lower post-traumatic stress disorder (PTSD)-related symptoms after the event [8, 9]. Family presence may lower litigation risks when family members believe that their child has been cared for with compassion and concern [10]. Given these observations, European and American medical associations [11–13] support FP during CPR. Even if FP during critical situations [14] or CPR in children is slowly becoming common [15], in only 41% of European countries family members are allowed to be present during in-hospital CPR for pediatric patients [16]. The main arguments include medico-legal concerns, potential healthcare professionals’ and parents’ stress, decreasing quality of care [17, 18], sterility issues, and space constraints [19]. However, family interference with the team in charge of the patient has not been reported to be a frequent issue during CPR [4, 20] trauma evaluation or emergency procedures [14]. For these reasons, in 2015, European Resuscitation Council Guidelines for Resuscitation promoted FP during resuscitation attempts [16]. Family presence might nonetheless be associated with negative experiences among healthcare professionals [19]. For this reason, the guidelines for Family-Centered Care written for the Neonatal, Pediatric, and Adult ICU support training for this practice [21]. The distribution of roles and the importance of a correct communication with parents during a critical situation or resuscitation attempts are part of the crisis resource management (CRM) and need, as well, to be trained during simulation. As a result, we set up an in situ simulation program aimed at effectively teaching PICU professionals the communication skills necessary to manage family presence during critical situations or resuscitation. The aim of this study was to evaluate the impact of this simulation program on healthcare professionals’ stress and the satisfaction.

## Materials and methods

### Setup: hybrid simulation

An already established in situ PICU simulation program (Simkids), which uses a human patient simulator (with high-technology mannequins: SimBabyClassic, NewSimBaby, and SimJunior; Laerdal Medical, Stavanger, Norway) in the PICU of Geneva Children’s Hospital, Switzerland, was modified by adding two actors (standard persons [SPs]) playing the parental role, to simulate family presence during CPR or other critical situation. A simulation can be defined as “in situ” when it is performed in the team’s usual work setting. Four different scenarios were set up: hemorrhagic shock; seizures; accidental extubation; and tricyclic acid intoxication (Supplemental Digital Content Document 1). Two simulation sessions per day were carried out. For each session, two groups participated in a randomly assigned scenario (one for each group). The team was exposed to a critical situation that sometimes might or might not progress to cardiac arrest and CPR. To focus on communication skills, the scenario and related medical algorithm were provided to the team in advance. Participants were usually represented by two physicians (one resident and one fellow), three nurses, and one nursing assistant. Each healthcare professional acted according to his/her role in the PICU. The following roles were assigned to participants: nurse in charge of the patient, nurse responsible for intravenous drugs preparation and administrations, and nurse in charge of patient’s parents. The nursing assistant might, as well, play the role of the family support person. Each actor received a summary containing the details of each clinical scenario beforehand. Pre-planned parental behavior (culpability, anger, despair, aggressiveness, physical and emotional breakdown) was performed by the actors (Supplemental Digital Content Document 1). In some scenarios, the actors deliberately disrupted patient care in an attempt to train participants’ ability to control external interference.

All healthcare professionals participated to the facilitator-guided post-event debriefing with good judgement [22]. Specific communication issues that might have arisen during the simulation were discussed with a trained psychologist and the PICU physician leading the simulation program. The SPs were invited to share their thoughts, feelings, and perceived level of support during the simulation. They shared suggestions for improvement based upon the received parental support at the bedside.

### Program evaluation

Participants were asked to complete a structured questionnaire created by the simulation team immediately after the debriefing sessions (Supplemental Digital Content Document 2) exploring three major topics: the experience of the healthcare professionals with parental presence before the implementation of this new simulation program; the self-perceived stress levels associated with parental presence before and after the simulation; and satisfaction after the simulation. A rating scale (0–10) was used for all responses. The participants also had to declare their healthcare professional category, age, gender, years of working experience after graduation, and years working in the PICU.

Some healthcare professionals participated in the simulation and answered the questionnaire twice. Since the questionnaire was completed anonymously, we were unable to locate these instances. Therefore, all questionnaires were analyzed together.

### Study outcomes

Primary outcomes were the difference in perceived stress level before and after the simulation, and the degree of satisfaction of the healthcare professionals. The impact of previous experience (none vs. > 1) on the field with family presence during CPR was evaluated by variation in perceived stress level.

### Statistical analysis

A paired *t*-test was performed to compare the level of stress before and after the simulation. A one-way ANOVA was used to explore potential associations between stress levels and demographic variables (i.e., healthcare professional category, age, gender, and working experience). *T*-test and Wilcoxon rank-sum test were used, as appropriate, to compare the variation in perceived stress levels (post- versus pre-simulation) between healthcare professionals who were previously exposed to real-life CPR with family presence versus those who were not. Continuous and categorical variables are presented as median (IQR). Stata software version 11 (StataCorp. 2009. Stata Statistical Software: Release 11. College Station, TX: StataCorp LP) was used for statistical analyses.

## Results

**Fig. 1:**
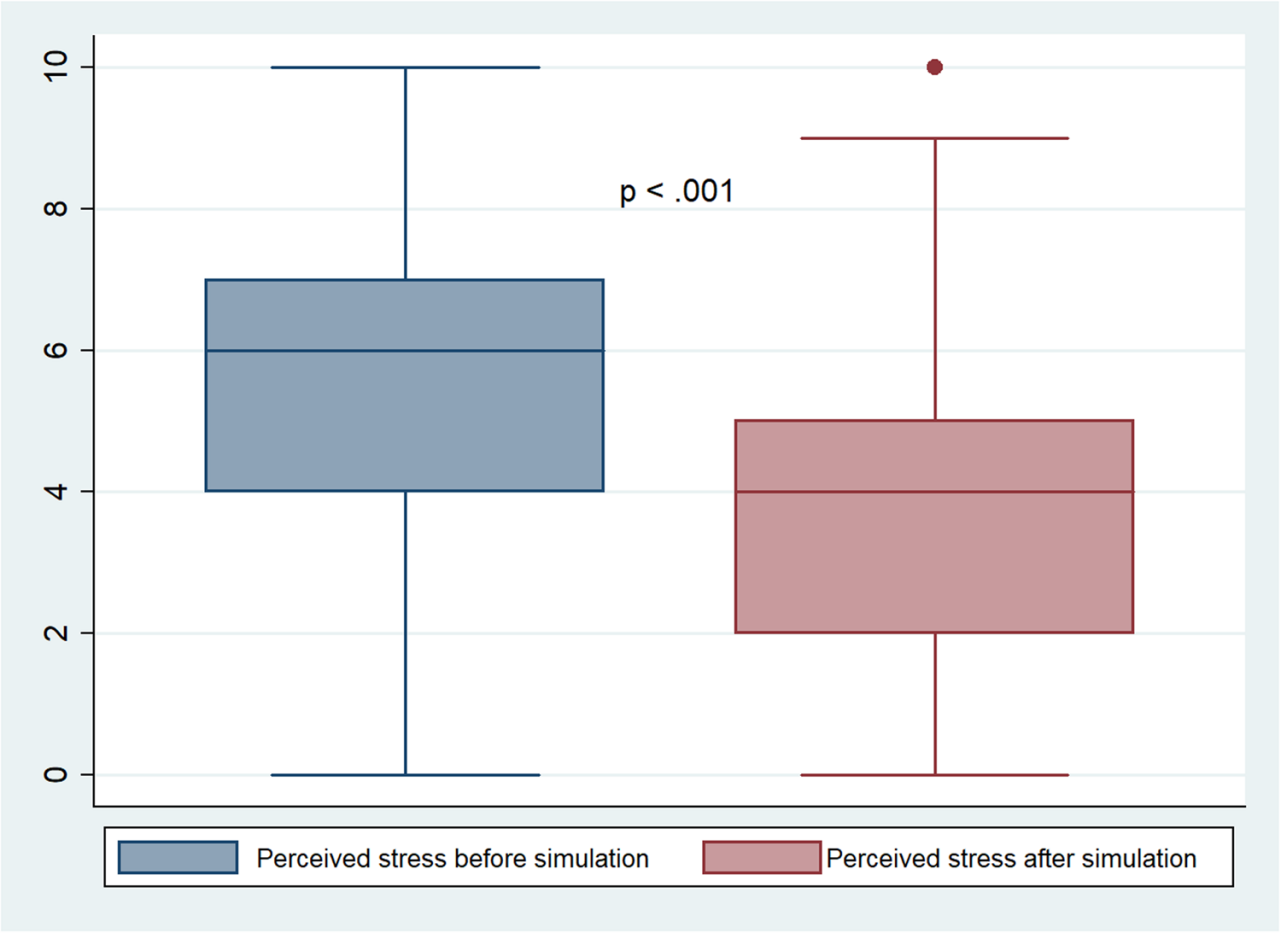
Box plot showing overall pediatric intensive care team stress related to family member’s presence during cardiopulmonary resuscitation or other major interventions in the pediatric intensive care unit, before and after the simulation

From January 2017 to January 2018, a total of 40 simulations were completed. Four different scenarios of simulation were played out. All participants agreed to fill in the survey. A total of 201 questionnaires were analyzed: 67 (33%) were completed by advanced practice registered nurses; 60 (30%) by registered nurses; 28 (14%) by assistant nurses; 44 (22%) by physicians, and 2 (1%) by medical students (Table 1). Perceived stress associated with parental presence decreased from a pre-simulation value of 6 (IQR, 4–7) to 4 (IQR, 2–5) post-simulation on a scale of 1–10 (Fig. 1, *p* < 0.0001). There was no association between self-perceived stress and healthcare professional category, age, gender, or working experience. The satisfaction rating of the participants after the simulation was high (median 10 out of 10). For 52 (25.7%) participants, the perceived post-simulation stress levels were higher than pre-simulation: 7 (IQR, 5–8) and 4 (IQR, 3–6), respectively (*p* < .001). There was no difference in perceived stress level variation between participants who had experienced critical situations or CPR with family presence at least once before and participants who did not.

**Table 1:** Characteristics of the pediatric intensive care healthcare professionals participants (*n* = 201)

**Role [*n*** **(%)]**	
Physician	44 (22)
Advanced practice registered nurse	67 (33)
Registered nurse	60 (30)
Nursing assistant	28 (14)
Médical student	2 (1)
**Demographic characteristics [*n*** **(%)]**	
Gender (woman)	179 (89)
Age (20–29 years)	71 (35)
Age (30–45 years)	124 (62)
Age (> 45 years)	6 (3)
**Working experience [years,** ***n*** **(%)]**	
0–5	56 (28)
> 6–10	64 (32)
> 11–15	39 (20)
> 16–20	30 (15)
> 21–25	5 (2)
> 25	6 (3)
**Number of CPR episodes with family presence in the past [*n*** **(%)]**	
0	38 (19)
1–5	129 (64)
6–10	34 (17)

*CPR* cardiopulmonary resuscitation

## Discussion

Our study describes an in situ pediatric simulation program of critical situations in the presence of family members played by professional actors. The simulation program was explicitly aiming at the development of communication skills. The “in situ” setting and the possibility to debrief in the presence of a psychologist represent two innovative features of our study. The simulation generally, but not always, showed a reduction in healthcare professionals perceived stress caused by family presence during a critical situation in the PICU. The overall satisfaction of the participants following the simulation was very high.

Our results are in line with a previous similar report on delivering bad news to patients and families in a pediatric emergency, where a simulation program with SPs and debriefing meetings improved fellows’ self-perceived comfort [23].

No association between self-perceived stress and healthcare professional category, or working experience was found in this study. These result are not in line with previous studies showing that nursing staff seem to be more supportive of FP than physicians [19, 25, 26]. Likewise, a previous study by Fein et al. suggests that practitioner level of experience may correlate with comfort with FP during invasive procedures [27]. In particular, attending physicians and nursing staff, presumably more experienced than residents, consistently supported FP during invasive procedures [27]. More senior staff such as attending physicians did not participate in our simulation, thus possibly explaining the discrepancy with our results.

There was no difference in perceived stress level variation between participants who had a previous experience with family presence during CPR and participants who had not. Experience is important but probably not sufficient to avoid healthcare professionals discomfort in the management of family presence during critical situations. Our results are in line with previous studies confirming that FP might negatively impact up to one-third of a group of experienced nurses [19].

In 25.7% of cases, the individually perceived stress level increased after simulation. We are not able to disentangle the contribution of family presence-related stress from the self-perceived stress related to the simulation itself. Moreover, different roles (communication with actors vs. patient care) might experience different levels of stress. The role of participants was not known. As a result, we are not able to explore the impact of the role played in the simulation in increasing post-simulation stress. This should be further investigated. In order to reduce the simulation-related stress as much as possible, a “debriefing with good judgement” approach was used, thus avoiding judgmental approach [22]. Participants’ confidentiality was maintained throughout. To meet the demand of participants experiencing a high level of stress after the simulation, a pre-simulation video on potential benefits and pitfalls of family presence during critical situations as well as on ad hoc communication tools is now available to all future participants.

The simulation sessions were run in the unit, and not in a simulation center as done by other studies [24]: the team has to learn how to effectively share the room space between healthcare professionals and family members, which is adapted to the actual degree of medical emergency with the eventual need for live-saving interventions. However, it is crucial that the healthcare professional in charge of the parents finds the right way to position them in the room, so as not to disturb the resuscitation team during CPR, or to allow the parents to be in close contact with their child if the clinical situation allows. The concern for space management around the patient’s bed might represent a barrier to the implementation of FP during resuscitation [19]. During training, we tried to highlight how important is that healthcare professionals should position parents in a way not to interfere with CPR attempts. We often noticed that a correct positioning of the parents was associated with a more harmonious and efficient management of the critical situation.

Another key component for success is the designation of a family support person, usually a seperate staff member, as it is advocated by medical associations [14, 28, 29]. Despite the fact that resuscitation’s team has to be absorbed in patient’s needs, the leader should identify the family support person at the beginning of the situation. ESPNIC recommends that it is the more experienced healthcare professionals who support the parents by providing a running commentary with appropriate explanations [13]. Social workers or clergy are usually designated as the family support person in north American PICU [14]. In our experience, the nursing assistants usually perform extremely well in this role, especially during the initial phases of the critical situation, when emotional support to families is key. Appropriate medical feedback is eventually conveyed by physicians and/or nurses once clinical stabilization is achieved.

European medical and nursing associations recommend that healthcare professionals explain to parents beforehand what to expect prior to entering the resuscitation area, provide a running commentary with appropriate explanations, help them to communicate their presence to their relative, respond truthfully and realistically to questions, maintain a safe environment, and assess continually their emotional and physical status [13]. A key role in the development of the aforementioned communication skills has been played by a psychologist who was present during the scenario, and led the debriefing to focus on the communication aspects. The psychologist highlighted the importance of nonverbal communication and helped the team cope with parents’ emotional breakdowns such as withdrawal, anger, or violence.

This study has limitations. First, we were not able to differentiate the contribution of the simulation self-perceived stress from family presence-related stress. We could not identify questionnaires from healthcare professionals who participated in the simulation more than once, thus potentially underestimating the effect of simulation on stress levels. Additional limitations are self-report measures and retrospective ranking of pre-stress level. Moreover, given the small sample size in many subgroups, association between self-perceived stress and healthcare category, age, gender, and experience may not have been present due to a lack of power. Also, we were not able to investigate the potential impact of the simulation role in the increase of post-simulation stress. A major limitation is represented by the fact that the questionnaire used in this study was created by the simulation group for the sake of this analysis and has never been validated.

## Conclusions

A simulation program seeking to provide skills focused on family presence management in the PICU might potentially reduce stress and was well accepted by participants. It might become an indispensable training intervention for the implementation of a PICU policy to allow family presence during CPR or critical situations. The increase in stress levels among some participants suggests a need to conduct this educational intervention in conjunction with other teaching modalities to improve healthcare professionals’ comfort.

## Supplementary information

Below is the link to the electronic supplementary material.

Supplementary file1 (DOCX 41 KB)

## Abbreviations

CPR: Cardiopulmonary resuscitation
CRM: Crisis resource management
ESPNIC: European Society of Paediatric and Neonatal Intensive Care
FP: Family presence
PICU: Pediatric intensive care unit
PTSD: Post-traumatic stress disorder
SP: Standard person
