# Supplementary material for: In situ simulation training for parental presence during critical situations in PICU: an observational study
Source: Eur J Pediatr. 2022 Mar 12;181(6):2409–14. doi: 10.1007/s00431-022-04425-8 (PMC9110492; doi:10.1007/s00431-022-04425-8)
Supplement: Supplementary file 1 — Supplementary file1 (DOCX 41 KB) [file 431_2022_4425_MOESM1_ESM.docx]

**Supplemental Document 1. Scenarios**

Each scenario represented a specific clinical situation: hemorrhagic shock due to a splenic fracture caused by a car accident; seizure caused by syndrome of inappropriate anti-diuretic hormone secretion (SIADH) in the setting of a pneumonia; accidental extubation; and tricyclic intoxication.

The standard person (SP) received information and preparation regarding the specific features of each clinical situation prior to the actual exercise. The expected actions and emotions (culpability, anger, despair, physical and emotional breakdown) of the SP during each training exercise were planned prior to the simulation event.

**Scenario 1:**

An 18-month-old girl was hospitalized in the pediatric intensive care unit (PICU) for pneumonia. She received non-invasive ventilation and antibiotics. On day 3, she presents seizures secondary to SIADH, with hyponatremia.

Scenography for SP:

A family with 3 children and married parents. The parents are present at the beginning of the simulation, each on one side of the bed. The mother alerts the nurse to unusual movement of her daughter. At the beginning the parents panic, but calm down if the team reacts appropriately. The parents are supportive of each other.

**Scenario 2:**

A 12-month-old boy was hospitalized in the PICU with a spleen fracture, following a road accident. He was in the mother’s car while she was driving to the supermarket. The car was traveling at 50 km/h, and crashed into a stopped car. The patient has been in the unit for 2 hours, and rapidly deteriorates. He presents with hemorrhagic shock by rupture of the spleen.

Scenography for SP:

The patient is the only child of a separated couple. The mother is present in the room at the beginning of the simulation, standing at the son’s bedside. She discussed her concerns regarding her culpability of the accident with the nurse. The father subsequently enters the room, and the patient has deteriorated. He is very worried about the condition of his son, and upset with the mother about her responsibility in the accident.

**Scenario 3**

A 3-month-old girl was hospitalized in the PICU for bronchiolitis. She was intubated on day 4. She presents with accidental extubation.

Scenography for SP:

The patient is the second child of a married couple. The father was holding his daughter in his arms, when a small movement caused extubation. The mother was seated across the bed. They are worried. The father feels guilty, and is in a total state of shock, standing close to the wall. The mother wants to stay close to her daughter and disturbs the team in charge of the patient. They do not support each other.

**Scenario 4**

A 15-month-old girl was in the PICU for monitoring of tricyclic intoxication. She ingested her mother’s pills, while her mom was watching the children. The patient develops rhythm disturbance with ventricular tachycardia.

Scenography for SP:

A family with 4 children, and married parents. The mother feels guilt, and is in the room at the beginning of the simulation. The father arrives later. The mother is shocked and the father stands too close to the bed, disturbing the team.


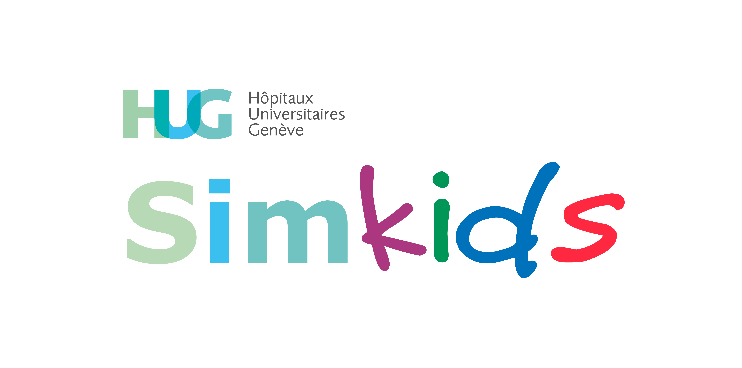
**Supplemental Document 2. Program Evaluation**

SimKids

Pediatric simulation program

Pediatric Department

Geneva University Hospitals

To add value in the training of caregivers in Family Members Presence during a resuscitation in the PICU, we would be grateful if you could answer this anonymous, multiple-choice, questionnaire.

1. How did you feel about your **level of stress related to the simulation before** the scenario?

□ □ □ □ □ □ □ □ □ □ □

0 1 2 3 4 5 6 7 8 9 10

No stress at all Maximum level of stress

1. How did you feel about the **level of stress related to parental presence in a resuscitation setting before** your participation to the scenario?

□ □ □ □ □ □ □ □ □ □ □

0 1 2 3 4 5 6 7 8 9 10

No stress at all Maximum level of stress

1. Which scenario did you take part in? (or join)

□ □

Scenario no. 1 Scenario no. 2

□ □

Observer in Scenario no.1 Observer in Scenario no. 2

1. What was your level of stress **at the end** of the scenario?

□ □ □ □ □ □ □ □ □ □ □

0 1 2 3 4 5 6 7 8 9 10

No stress at all Maximum level of stress

1. How many resuscitations have you attended before this simulation in the presence of parents or family?

□ □ □ □ □ □ □ □ □ □ □

0 1 2 3 4 5 6 7 8 9 10+

1. Do you think that it would be useful to carry out training concerning parental presence in the setting of a resuscitation?

□ □ □ □ □ □ □ □ □ □ □

0 1 2 3 4 5 6 7 8 9 10

Not useful at all Very useful

1. Are you satisfied with your participation to this situation?

□ □ □ □ □ □ □ □ □ □ □

0 1 2 3 4 5 6 7 8 9 10

Not satisfied at all Really satisfied

1. What is your profession?

| □ Physician |
| --- |
| □ Advanced practice registered nurse |
| □ Registered nurse |
| □ assistant nurse |

□ Other: ___________

1. Are you?

□ □

A woman A man

1. Which age category do you belong to?

□ □ □ □ □

20-25 y.o. 26-30 y.o. 31-35 y.o. 36-40 y.o. 41-45 y.o.

□ □ □ □

46-50 y.o. 51-55 y.o. 56-60 y.o. 61-65 y.o.

1. What is your professional experience (after your graduation)?

□ □ □ □ □

0-5 years 6-10 years 11-15 years 16-20 years 21-25 years

□ □ □ □

26-30 years 31-35 years 36-40 years >41 years

1. How long have you been working in the PICU?

□ □ □ □ □

0-5 years 6-10 years 11-15 years 16-20 years 21-25 years

□ □ □ □

26-30 years 31-35 years 36-40 years >41 years
